# Synthesis of 2-Ethylhexyl 5-Bromothiophene-2-Carboxylates; Antibacterial Activities against *Salmonella* Typhi, Validation via Docking Studies, Pharmacokinetics, and Structural Features Determination through DFT

**DOI:** 10.3390/molecules29133005

**Published:** 2024-06-25

**Authors:** Waseem Nazeer, Muhammad Usman Qamar, Nasir Rasool, Mohamed Taibi, Ahmad Mohammad Salamatullah

**Affiliations:** 1 Department of Chemistry, Government College University, Faisalabad 38000, Pakistan; waseemgondal78@gmail.com; 2 Institute of Microbiology, Government College University, Faisalabad 38000, Pakistan; musmanqamar@gcuf.edu.pk; 3 Division of Infectious Disease, Department of Medicine, University of Geneva, 1206 Geneva, Switzerland; 4 Laboratory of Therapeutic and Organic Chemistry, Faculty of Pharmacy, University of Montpellier, 34000 Montpellier, France; mohamedtaibi9@hotmail.fr; 5 Department of Food Science and Nutrition, College of Food and Agricultural Sciences, King Saud University, P.O. Box 2460, Riyadh 11451, Saudi Arabia; asalamh@ksu.edu.sa

**Keywords:** 2-ethylhexyl 5-bromothiophene-2-carboxylate, Suzuki, XDR *Salmonella* Typhi, antibacterial, molecular docking, density functional theory

## Abstract

A new class of thiophene-based molecules of 5-bromothiophene-2-carboxylic acid (**1**) have been synthesized in current research work. All analogs **4A**–**4G** were synthesized with optimized conditions by coupling reactions of 2-ethylhexyl 5-bromothiophene-2-carboxylate (**3**) with various arylboronic acids. The results indicated that the majority of compounds showed promising effective in vitro antibacterial activity. Herein, 2-ethylhexyl-5-(p-tolyl)thiophene-2-carboxylate (**4F**), in particular among the synthesized analogs, showed outstanding antibacterial action (MIC value 3.125 mg/mL) against XDR *Salmonella* Typhi compared to ciprofloxacin and ceftriaxone. The intermolecular interaction was investigated by using a molecular docking study of thiophene derivatives **4A**–**4G** against XDR *S*. Typhi. The values of the binding affinity of functionalized thiophene molecules and ciprofloxacin were compared against bacterial enzyme PDB ID: 5ztj. Therefore, **4F** appears to be a promising antibacterial agent and showed the highest potential value. Density functional theory (DFT) calculations were executed to examine the electronic, structural, and spectroscopic features of the newly synthesized molecules **4A**–**4G**.

## 1. Introduction

The public health risk of sepsis is increasing, particularly in underdeveloped countries where it is a syndromic response to infections. The World Health Organization (WHO) revealed that there have been 48.9 million reported cases of sepsis, and it has been reported that one person dies globally every 2.58 s. Twenty million cases were also discovered in children, with 2.9 million deaths worldwide—85% of which occurred in developing countries. *Salmonella* Typhi, the same bacteria that causes typhoid fever, is one of the leading causes of pediatric sepsis in typhoid-endemic areas [1]. Extensively drug-resistant *S*. Typhi (XDR *S*. Typhi) is the cause of over 90% of recorded cases of typhoid disease and is more common in younger patients. However, most studies on antibiotic resistance to date have employed isolates of *S*. Typhi from adult patients. These XDR *S*. Typhi strains have spread throughout the world, with reports from the USA, UK, India, Australia, and Bangladesh [2]. As a result, there has been increasing concern regarding the prevalence of XDR *S*. Typhi strains are insusceptible to chloramphenicol, ampicillin, trimethoprim, ciprofloxacin, and ceftriaxone [3]. In nature, heterocyclic compounds are used for plenty of synthetic and biological purposes. Thiophenes are a class of sulfur-based heterocyclic compounds that may have therapeutic effects on a variety of infections. Thiophene-based compounds can be made quickly and have biological functions, which has provoked a lot of interest in their synthesis [4,5,6,7,8,9,10,11,12,13]. Their biological outcomes comprise antifungal, antiviral, antibacterial, anti-inflammatory, and cytotoxic characteristics [14,15]. Harit et al. reported the antibacterial efficacy of thiophene-based compounds against *Staphylococcus aureus*, *Escherichia coli*, and *Salmonella* enterica subsp. enterica serovar Typhimurium [16], and 1,1′-(2,5-thiophenediyl)bis [1-(2-benzofuranyl) methanone] with the highest MIC value of 32 µg m/L has shown good efficacy against *S. aureus* and *E. coli* [17]. In particular, our research group has described the synthesis and biological importance of several moderate-to-good yield thiophene-based derivatives that demonstrate antibacterial activity against *E. coli*. In addition, 2,5-bis(4-chlorophenyl)-3-hexylthiophene and 2-bromo-5-(3-chloro-4-fluorophenyl)-3-hexylthiophene revealed the highest biofilm inhibition activity against *E. coli*. [18,19]. Regarding the importance of thiophene-based functionality for biological processes, our research team intended to design and synthesize some thiophene-based molecules and trial them for antibacterial efficacy against XDR *S*. Typhi. Therefore, we reveal herein a simple method of synthesizing thiophene-based molecules with good yields in the quest for new bioactive molecules. The goal of the current research was to use 2-ethylhexyl 5-bromothiophene-2-carboxylate and various arylboronic acids in Suzuki cross-coupling procedures to make several thiophene analogs. For biological characterization, all of these compounds underwent screening for potential XDR *S*. Typhi. The most effective compounds were selected through a theoretical study using molecular docking. DFT was used to examine the structural, spectroscopic, and electrical characteristics of the synthesized thiophene-based molecules to evaluate the structure stability and reactivity.

## 2. Results and Discussion

### 2.1. Chemistry

**Scheme 1:**
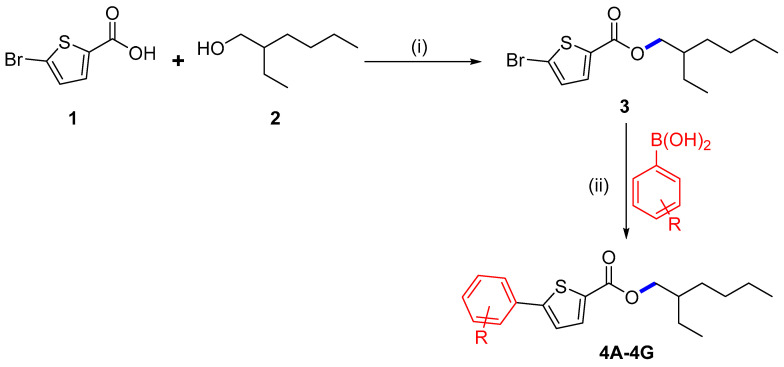
Synthesis of **3**: (i) **1** (2 g, 9.65 mmol), DCM (100 mL), 2-ethyl hexanol (1.25 g, 9.65 mmol), DCC (1.99 g, 9.65 mmol), DMAP (0.23 g, 1.93 mmol), 30 °C. (ii) Synthesis of derivatives **4A**–**4G**, 1,4-dioxane/water (6 mL), **3** (0.15 g, 0.46 mmol), aryl boronic acid (1.1 eq), Pd(PPh_3_)_4_ (5 mol%), K_3_PO_4_ (2 eq), 90 °C, 16 h reflux.

In the current study, we began with commercially available 5-bromothiophene-2-carboxylic acid (**1**), which was initially esterified using the scheme formerly described [20,21]. Compound **1** was reacted with 2-ethyl hexanol (**2**) to synthesize 2-ethylhexyl 5-bromothiophene-2-carboxylate (**3**) with a 72% yield (Scheme 1). Herein, DCC (N,N′-dicyclohexylcarbodiimide) attended as the coupling agent, and DMAP (dimethylaminopyridine) assisted as the catalyst in the above-mentioned process with dichloromethane (DCM) as a solvent stirred at 30 °C.

**Scheme 2:**
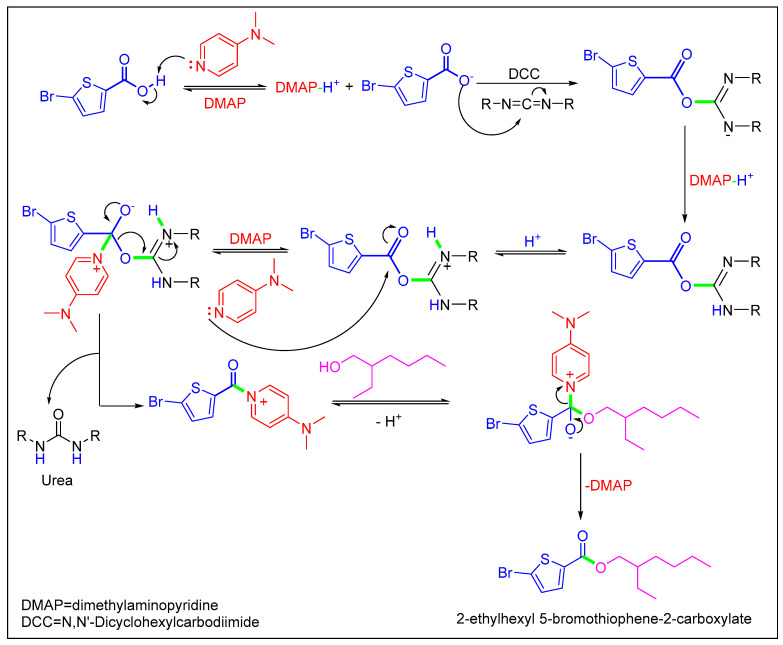
Mechanistic pathway of Stiglich esterification of 5-bromothiophene-2-carboxylic acid.

**Scheme 3:**
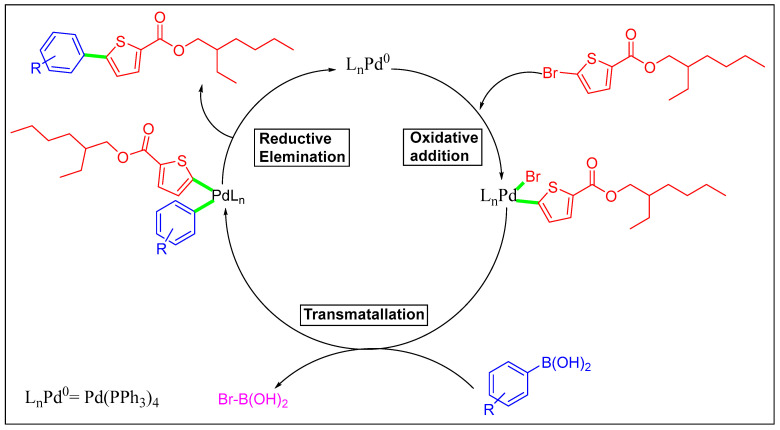
Mechanistic pathway of Suzuki cross-coupling of 2-ethylhexyl 5-bromothiophene-2-carboxylate.

We planned an easy method under ideal circumstances to explore the Suzuki cross-coupling of **3**, which was palladium-catalyzed. We concentrated on the synthesis of new thiophene derivatives by using Pd(PPh_3_)_4_ as a catalyst for the Suzuki reaction at 90 °C. The derivatives of 2-ethylhexyl 5-bromothiophene-2-carboxylate **4A**–**4G** were successfully synthesized by reacting **3** with different arylboronic acids. Herein, we conducted these couplings in 1,4-dioxane as a solvent and water ratio of 6:1; freshly synthesized thiophene molecules **4A**–**4G** were produced in 60.1–79.1% yields (Scheme 1, Table 1). The maximum solvability of the aryl boronic acid in 1,4-dioxane and water could be the root of the higher yield [18,19]. The maximum and minimum yield of thiophene analogs based on electron withdrawing and electron donating effects are also noteworthy [22]. It was noted that **4A** showed a maximum yield (79.1%) with the electron-donating group while **4D** showed a minimum yield (60.7%) due to the existence of an electron-withdrawing group among the thiophene analogs (Scheme 2 and Scheme 3).

### 2.2. Antibacterial Activity of the Antibiotics and Compounds (**4A**–**4G**) against XDR S. Typhi

XDR *S*. Typhi is resistant to medically important antibiotics including chloramphenicol, ampicillin, co-trimoxazole, ceftriaxone, and ciprofloxacin, and is sensitive to azithromycin, imipenem, and meropenem. The agar well diffusion method was used to test the thiophene derivatives **4A**–**4G** for in vitro antibacterial activity compared to *Salmonella* Typhi at five dissimilar concentrations (10, 20, 30, 40, and 50 mg/well) based on the results of molecular docking and DFT. By associating the regular diameter of the bacterial growth inhibition zones surrounding the well (measured in mm) with ciprofloxacin (an antibiotic control), it is possible to assess how susceptible certain microbial isolates are to the test chemicals. According to the data listed in Table 2, the inhibition zone rose as the concentration of the synthesized molecules **4A**–**4G** increased. Compound **4F** displayed the largest zone of inhibition, measuring 25 mm at a dosage of 50 mg/mL. Compound **4F** had values of 3.125 mg MIC and 6.25 mg MBC. Compounds **4A** and **4D** also exhibited inhibition against XDR *S.* Typhi. Ciprofloxacin and ceftriaxone antibiotics were used as positive controls, where XDR *S*. Typhi was resistant to both antibiotics, as shown in Table 2 and Table 3.

### 2.3. Structure–Activity Relationship (SAR)

The following structural–activity relationship assumptions were suggested based on the experimental results of the antibacterial activity of the synthesized compounds.

- In this research, among the synthesized thiophenes, molecule **4F** inhibited the clinical isolate XDR *S*. Typhi at an MIC 3.125 mg/mL.
- It is interesting to note that the remarkable antibacterial activity of **4F** against XDR *S*. Typhi is due to the presence of dual thiophene moieties.

### 2.4. Theoretical Analysis

#### 2.4.1. Molecular Docking Studies

**Figure 1:**
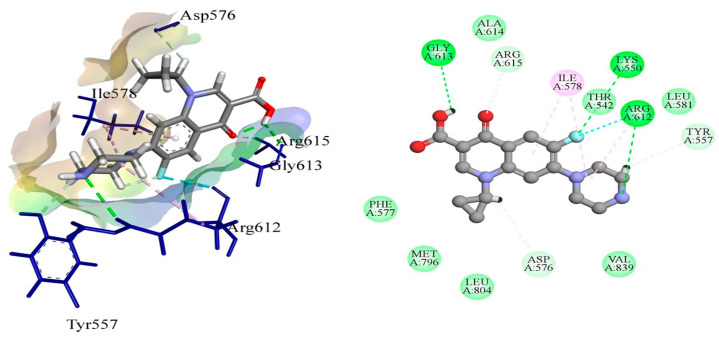
The putative binding mode of ciprofloxacin within the active pocket of DNA gyrase protein PDB ID: 5ztj.

**Figure 2:**
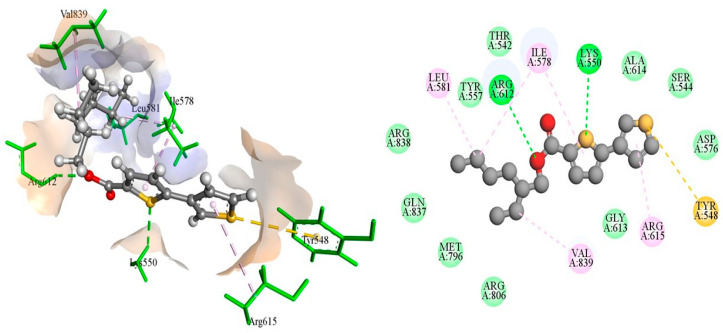
The putative binding mode of 4F within the active pocket of DNA gyrase protein PDB ID: 5ztj.

The DNA gyrase subunit A protein crystal structure with PDB ID: 5ztj has a single chain of 312 amino acids. This crystal structure has a resolution of 2.4 Å. DNA gyrase is essential in cellular processes; therefore, it serves as an ideal drug target for the treatment of *Salmonella* Typhi-induced infections. Ciprofloxacin is a reference standard antibiotic that specifically inhibits DNA gyrase. Therefore, it was used to compare the inhibitory potential of newly produced molecules. Ciprofloxacin was found to have a high binding affinity in the predicted active pocket of DNA gyrase with a binding energy of ΔG = −5.61799 Kcal/mole. It develops three hydrogen bonds with Lys550, Arg612, and Gly613, whereas Ile578 and Arg615 are linked to ciprofloxacin through C–H interactions. Alkyl and π-alkyl interactions were also established with Ile578 and Arg615, as presented in Figure 1. The most potent compound among these synthesized compounds was found to be **4F**, which had a binding energy of −7.38549 KCal/mole. This molecule established two hydrogen bonds with amino acid residues of Lys550 and Arg612. A π-sulfur interaction was also found with Tyr548 in the active pocket and four alkyl and π-alkyl interactions were also found with Ile578, Leu581, Arg615, and Val839, as presented in Figure 2 and Table 4.

#### 2.4.2. Drug Likeness and Pharmacokinetics

**Figure 3:**
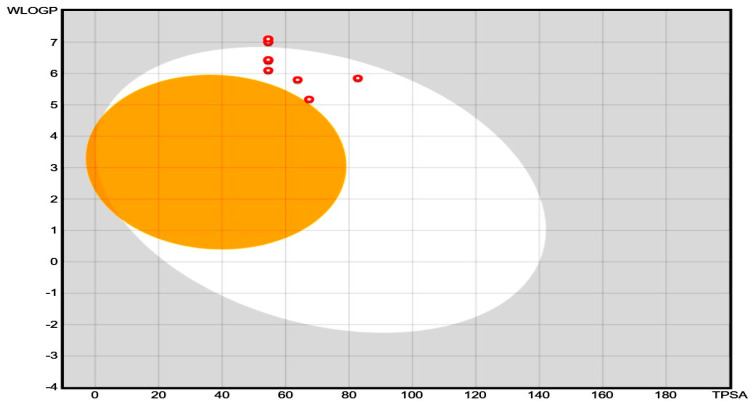
Boiled egg plot for 2-ethylhexyl 5-bromothiophene-2-carboxylate’s derivatives (**4A**–**4G**) for ADME evaluation.

The pharmacokinetic properties (e.g., absorption, distribution, metabolism, excretion, also called ADME properties) and drug-likeness of the synthesized compound were predicted by using SwissADME, which is a free web tool to determine the ADMET properties and drug ability of small molecular weight compounds. This software uses certain algorithms to predict the ADME values of newly synthesized compounds, and the consequences are shown in Table 5. The consequences of Table 5 are shown as a boiled egg plot in Figure 3 to better represent the gastrointestinal permeability. The majority of compounds exhibited good GIT absorption, and none of these molecules could cross the blood-brain barrier (BBB). The synthesized molecules were also found to fulfill the drug ability criteria. Therefore, these molecules could serve as potential drug candidates. The ADME studies of compounds **4A**, **4B**, **4C**, **4D, 4E**, **4F**, and **4G** were performed through in silico-based methods, and the predicted pharmacokinetic values are listed in Table 5. These values provide valuable information about the GI absorption and ability of synthesized compounds to cross the blood-brain barrier (BBB). The boiled egg plot in Figure 3 (generated by the SwissADME web server) represents two parts: the outer whitish part shows that the compound can easily be absorbed from the GIT, and the inner yellowish part shows that the compounds can cross the BBB. The synthesized compounds appeared in the outer whitish portion, which indicates that these compounds can be easily absorbed from the GI and cannot cross the BBB, which is a characteristic feature of these newly synthesized compounds to reduce CNS side effects. Moreover, the compounds were further evaluated for drug ability and through the PAINS filter, where the results showed that none of the synthesized compounds violated the PAINS criteria, which shows that the structures of the newly synthesized compounds were unique [23]. Similarly, most of these synthesized compounds followed the Lipinski rule of five for drug ability [24]. All compounds (**4A** to **4G**) showed a maximum total polar surface area between 54.54 Å and 82.78 Å, therefore displaying a high predicted GIT absorption [25]. Therefore, considering all of these parameters, these compounds could potentially be oral drug candidates for further optimization.

### 2.5. DFT Studies

The noteworthy progress made through computational chemistry means that DFT has been extensively used on many scientific grounds, especially in the analysis of reaction mechanisms. These investigations entail a multitude of intricate calculations, typically conducted through a Gaussian 09 software program. The research of input files and the analysis of output files are not easy tasks, generally concerning laborious and complicated steps. All of the synthesized compounds (**4A**–**4G**) were computed at the PBE1PBE/def2svp level of theory [26]. The computation was accomplished using GAUSSIAN 09W, with all outcomes visualized through Gauss View. Numerous properties including optimized geometry, frontier molecular orbitals, and energy gap were determined. Furthermore, the anticipation of molecular reactivity and chemical behavior was conducted operating both local and global expressive parameters. The calculation included determining the global reactivity framework such as electron affinity, ionization energy, chemical softness and hardness, electrophilicity, and chemical potential. Chemical hardness reflects the capacity to withstand alterations in electron distribution, thereby influencing the solidity and reactivity of compounds, while chemical softness is contrarywise correlated with hardness, indicating flexibility. Electronegativity denotes the propensity of molecules to attract electrons, while its negativity is mentioned as electron chemical potential. Furthermore, when the chemical system accepts an external charge, the electrophilic index can serve as a measure of energy stability. The 3D optimized geometries of all molecules planned at the PBE1PBE/def2svp level of theory are given in the Supplementary Materials (Figure S12). In 3D models, brown represents hydrogen, the yellow color denotes sulfur, the pink color symbolizes carbon, the cyan color indicates oxygen, the blue color is chlorine, and the purple color represents nitrogen.

#### 2.5.1. NMR Spectra

Organic chemists utilize nuclear magnetic resonance (NMR) to check the structure of the produced molecules. DFT computation of NMR chemical shifts can produce satisfactory NMR datasets for comparison with fact-finding datasets, hence, an increasing trust in NMR. NMR values were conducted at the same theoretical level as optimization for all of the simulated molecules and then related to the experimental chemical shifts. The Boltzmann-averaged ^1^H-NMR chemical shifts for compounds **4A**–**4G** are given in the Supplementary Materials (Tables S1–S7). The remaining compounds are specified in the supporting data; they were in good arrangement with the experimental chemical shifts. As a result, the NMR data of the molecules that could not be synthesized in a decent yield to acquire their experimental NMR were predicted with high assurance and can be used as the basis for the future synthesis of these molecules.

#### 2.5.2. Frontier Molecular Orbital (FMO) Analysis

**Figure 4:**
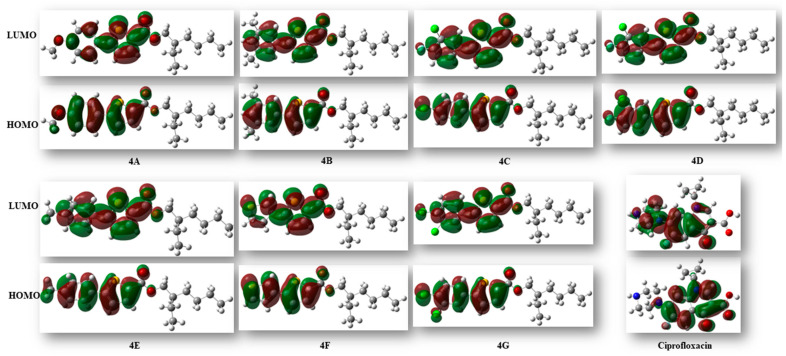
Isosurface illustration of the HOMO and LUMO of all the synthesized molecules and ciprofloxacin.

The reactivity and various properties of compounds can be obtained through FMO analysis. The disparity in energy between the highest occupied molecular orbital (HOMO) and the lowest unoccupied molecular orbital (LUMO) offers insights into the overall reactivity of molecules. When the HOMO–LUMO energy differences are high, the reactivity of the compound is less, and vice versa [27]. The computed energy difference of HOMO and LUMO of all the derivatives was observed to be in the range of 3.98–4.69 eV. In this series, compound **4D**, comprised of pyrimidine and a thiophene ring, exhibited the largest HOMO–LUMO gap at 4.69 eV and showed the highest stability and lowest reactivity compared to the others. The energies of HOMO, LUMO, and the gap between HOMO and LUMO are given in Table 6 (Figure 4).

#### 2.5.3. MESP

**Figure 5:**
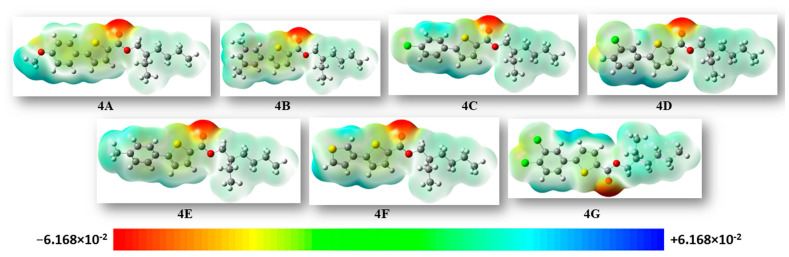
For all of the synthesized molecules, the MEP maps were plotted using an iso value of 0.0004. Units of the scale of electron charge density are in Hartree.

The MESP (molecular electrostatic potential surface) concurrently exhibits the shape and electrostatic potential values by plotting the electrostatic potential mapped on the iso-electron density surface [28]. The molecular electrostatic potential was plotted for the compounds and is illustrated in Figure 5. The mapping of the molecular electrostatic potential has been proven to be highly beneficial in the study of the physicochemical properties of the developed molecules. Various colors on the surface indicate different electrostatic potential values: red indicates the most electronegative potential areas, blue indicates the utmost positive electrostatic potential areas, and green indicates areas with zero potential. The bioreactivity of a molecule can also be analyzed through the electrostatic interactions occurring between the receptor-active sites and that molecule. As depicted in Figure 5, negative regions (shown in red) were predominantly localized over the oxygen atoms of the ester group, expecting these sites to be the maximum reactive positions for electrophilic attack. On the other hand, the extremely positive areas (blue) were localized over the hydrogen atoms of the thiophene and benzene rings, revealing that they were the most reactive for nucleophilic attack.

#### 2.5.4. Conceptual DFT Reactivity Descriptors

The reactivity of chemical compounds can be described by certain electronic descriptors like chemical hardness (*η*), electron affinity (*A*), ionization potential (*I*), and electronic chemical potential (*μ*). These properties can easily be calculated by DFT computations. The determination of *I* and *A* are grounded in Koopman’s theorem, which stipulates that the negative of EHOMO and ELUMO corresponds to the electron affinity (*A*) and ionization potential (*I*) of the molecules. The other descriptors (i.e., *η*, *µ*, and *ω*) are then calculated as follows:

*η* = (E_LUMO_ − E_HOMO_)/2 (1)

*μ* = (E_LUMO_ + E_HOMO_)/2 (2)

*ω* = *μ*^2^/2*η* (3)

Table 7 presents the standards of all the significant reactivity signifiers for the molecules under investigation. A negative chemical potential signifies compound stability. The hardness denotes the molecule’s resistance to electron cloud deformation during the chemical processes. Hard systems are relatively compact with low polarizability, while soft systems are larger and highly polarizable. Calculations indicated the system’s relative softness, likely attributed to the presence of two S-atoms.

## 3. Materials and Methods

### 3.1. Information on the Compounds

A melting point gadget B-540 was employed to figure out the melting points of the freshly synthesized molecules. Shanghai *Macklin Biochemical* provided the chemicals and reagents of analytical quality. The synthesized products were subjected to measurement of their ^1^H NMR spectra using an Avance III Bruker spectrometer operating at 400 MHz with chloroform as the solvent. Parts per million (ppm) were used to represent the chemical shift readings, whereas Hertz was used to estimate the coupling constant values. The percentage of elements (C, H, S) was investigated by using an elemental analyzer (EURO EA 3000). Silica gel column chromatography was employed for chemical purification. Verification of reaction completion was conducted via thin-layer chromatography (TLC) using PF254-coated cards sourced from Merck, Germany with a pore size of 60 Å. The detection of freshly synthesized thiophene derivatives was facilitated by a UV lamp releasing light in the array of 254 to 365 nm to find the spots.

### 3.2. Synthesis of the 2-Ethylhexyl 5-Bromothiophene-2-Carboxylate (**3**)

A weighed portion of **1** (2 g, 9.65 mmol), along with 100 mL of dry DCM and a magnetic stirrer was added to a dry flask (250 mL). In addition, DMAP (0.23 g, 1.93 mmol) and 2-ethyl hexanol (1.25 g, 9.65 mmol) were added to the aforementioned blend while keeping the temperature at 0 °C in isotherm. The coupling reagent DCC (1.99 g, 9.65 mmol) was then added and stirred for 10 min. After one hour, the flask was detached from the isotherm and placed at standard temperature. After 6 h of stirring, the mixture was separated and a rotary evaporator was used for the solvent evaporation. The basic product was cleaned via column chromatography with n-hexane even as the solvent [20,21]. Finally, the structures of the freshly synthesized molecules were determined using NMR techniques.

### 3.3. Synthesis of Compounds **4A**–**4G**

At room temperature, the Pd(PPh_3_)_4_ catalyst (5 mol%), 1,4-dioxane as the solvent (6 mL), and **3** (0.15 g, 0.47 mmol) were added to a dehydrated Schlenk tube and agitated for 30 min under argon. Arylboronic acid (1.1 eq) and the base K_3_PO_4_ (2 eq) were added to the above-mentioned blend. This was then refluxed for approximately 16 h at 90 °C under an inert atmosphere. The completion of the reaction was verified via TLC. Subsequently, the blend underwent filtration and concentration by evaporating the solvent. Synthesized molecules were cleaned via column chromatography with ratios of n-hexane to ethyl acetate **4A**–**4G**, [29,30]. The newly synthesized compounds were examined using NMR spectroscopy.

### 3.4. Characterization Data

2-ethylhexyl 5-(4-methoxyphenyl)thiophene-2-carboxylate (**4A**). Starting with **3** (0.15 g, 0.47 mmol), Pd(PPh_3_)_4_ catalyst (0.027 g, 2.34 mmol), K_3_PO_4_ (0.199 g, 0.94 mmol), and 4-MeOC_6_H_4_B(OH)_2_ (0.078 g, 0.51 mmol), **4A** was isolated as a colorless solid (79.1%). ^1^H NMR (400 MHz, chloroform) δ 7.75 (d, *J* = 4.2 Hz, 1H), 7.65–7.53 (m, 2H), 7.20 (dd, *J* = 4.0, 1.0 Hz, 1H), 7.04–6.90 (m, 2H), 4.24 (dd, *J* = 5.7, 2.9 Hz, 2H), 3.92 (s, 1H), 3.87 (s, 3H), 1.73 (p, *J* = 6.0 Hz, 1H), 1.57 (s, 4H), 1.51–1.26 (m, 8H), 1.01–0.90 (m, 5H). Analysis calculated for C_20_H_26_O_3_S: C, 69.33; H, 7.56; S, 9.25. Found; C, 69.31; H, 7.53; S, 9.24%.

2-ethylhexyl 5-(3,5-dimethylphenyl)thiophene-2-carboxylate (**4B**). Starting with **3** (0.15 g, 0.47 mmol), Pd(PPh_3_)_4_ catalyst (0.027 g, 2.35 mmol), K_3_PO_4_ (0.199 g, 0.94 mmol), and 3,5-Me_2_C_6_H_3_B(OH)_2_ (0.076 g, 0.51 mmol), **4B** was found as a brown solid (74.4%). ^1^H NMR (400 MHz, chloroform) δ 7.83 (d, *J* = 6.8 Hz, 1H), 7.37 (d, *J* = 2.1 Hz, 2H), 7.24 (d, *J* = 6.7 Hz, 1H), 6.96 (t, *J* = 2.1 Hz, 1H), 4.24 (t, *J* = 6.1 Hz, 2H), 2.30 (s, 6H), 1.75 (tt, *J* = 7.4, 6.1 Hz, 2H), 1.53–1.45 (m, 2H), 1.39–1.27 (m, 4H), 0.89 (t, *J* = 6.8 Hz, 3H). Analysis calculated for C_21_H_28_O_2_S: C, 73.21; H, 8.19; S, 9.31. Found; C, 73.23; H, 8.17; S, 9.32%.

2-ethylhexyl 5-(4-chlorophenyl)thiophene-2-carboxylate (**4C**). Starting with **3** (0.15 g, 0.47 mmol), Pd(PPh_3_)_4_ catalyst (0.027 g, 2.34 mmol), K_3_PO_4_ (0.199 g, 0.94 mmol), and 4-ClC_6_H_4_B(OH)_2_ (0.079 g, 0.51 mmol), **4C** was separated as a white solid (65.1%). ^1^H NMR (400 MHz, chloroform) δ 7.83 (d, *J* = 6.7 Hz, 1H), 7.76–7.61 (m, 3H), 7.48–7.35 (m, 2H), 4.24 (t, *J* = 6.1 Hz, 2H), 1.75 (tt, *J* = 7.3, 6.1 Hz, 2H), 1.49 (dq, *J* = 7.9, 7.0 Hz, 2H), 1.41–1.24 (m, 4H), 0.89 (t, *J* = 6.8 Hz, 3H). Analysis calculated for C_19_H_23_ClO_2_S: C, 65.04; H, 6.61; S, 9.14. Found; C, 65.05; H, 6.60; S, 9.13%.

2-ethylhexyl 5-(3-chloro-4-fluorophenyl) thiophene-2-carboxylate (4D). Starting with **3** (0.15 g, 0.47 mmol), Pd(PPh_3_)_4_ catalyst (0.027 g, 2.34 mmol), K_3_PO_4_ (0.199 g, 0.94 mmol), and 3-Cl,4-FC_6_H_3_B(OH)_2_ (0.088 g, 0.51 mmol), **4D** was found as a brown solid (60.7%). ^1^H NMR (400 MHz, chloroform) δ 7.77 (d, *J* = 3.9 Hz, 1H), 7.69 (dd, *J* = 6.8, 2.3 Hz, 1H), 7.51 (ddd, *J* = 8.5, 4.5, 2.3 Hz, 1H), 7.26–7.17 (m, 2H), 4.41–4.11 (m, 2H), 1.73 (p, *J* = 6.1 Hz, 1H), 1.49–1.34 (m, 8H), 1.03–0.90 (m, 7H). Analysis calculated for C_19_H_22_ClFO_2_S: C, 61.86; H, 6.01; S, 8.69. Found; C, 61.85; H, 6.02; S, 8.66%.

2-ethylhexyl 5-(*p*-tolyl)thiophene-2-carboxylate (**4E**). Starting with **3** (0.15 g, 0.47 mmol), Pd(PPh_3_)_4_ catalyst (0.027 g, 2.34 mmol), K_3_PO_4_ (0.199 g, 0.94 mmol) and 4-MeC_6_H_4_B(OH)_2_ (0.069 g, 0.51 mmol), **4E** was isolated as a white solid (72.6%). ^1^H NMR (400 MHz, chloroform) δ 7.77 (t, *J* = 3.7 Hz, 1H), 7.56 (dd, *J* = 8.3, 2.3 Hz, 2H), 7.28–7.22 (m, 3H), 4.32–4.14 (m, 2H), 3.92 (s, 1H), 2.41 (s, 3H), 1.83–1.59 (m, 1H), 1.53–1.25 (m, 5H), 0.96 (dt, *J* = 14.5, 7.2 Hz, 4H). Analysis calculated for C_20_H_26_O_2_S: C, 72.69; H, 7.93; S, 9.70. Found; C, 72.67; H, 7.91; S, 9.67%.

2-ethylhexyl [2,3′-bithiophene]-5-carboxylate (**4F**). Starting with **3** (0.15 g, 0.47 mmol), Pd(PPh_3_)_4_ catalyst (0.027 g, 2.34 mmol), K_3_PO_4_ (0.199 g, 0.94 mmol), and C_4_H_3_B(OH)_2_S (0.065 g, 0.51 mmol), **4F** was separated as an off-white solid (69.3%). ^1^H NMR (400 MHz, chloroform) δ 7.74 (dd, *J* = 4.6, 3.9 Hz, 1H), 7.53 (dd, *J* = 2.9, 1.4 Hz, 1H), 7.41 (ddd, *J* = 5.1, 2.8, 1.1 Hz, 1H), 7.35 (dt, *J* = 5.0, 1.5 Hz, 1H), 7.20 (dd, *J* = 3.9, 1.1 Hz, 1H), 4.24 (dd, *J* = 5.8, 2.7 Hz, 1H), 3.92 (s, 2H), 1.77–1.57 (m, 1H), 1.54–1.28 (m, 6H), 0.96 (dt, *J* = 14.3, 7.0 Hz, 6H). Analysis calculated for C_17_H_22_O_2_S_2_: C, 63.32; H, 6.88; S, 19.88. Found; C, 63.33; H, 6.85; S, 19.87%.

2-ethylhexyl 5-(3,4-dichlorophenyl)thiophene-2-carboxylate (**4G**). Starting with **3** (0.15 g, 0.47 mmol), Pd(PPh_3_)_4_ catalyst (0.027 g, 2.34 mmol), K_3_PO_4_ (0.199 g, 0.94 mmol), and 4-MeC_6_H_4_B(OH)_2_ (0.099 g, 0.51 mmol), **4G** was separated as a dark brown solid (62.7%). ^1^H NMR (400 MHz, chloroform) δ 7.75 (d, *J* = 4.2 Hz, 1H), 7.65–7.53 (m, 2H), 7.20 (dd, *J* = 4.0, 1.0 Hz, 1H), 7.04–6.90 (m, 2H), 4.24 (dd, *J* = 5.7, 2.9 Hz, 2H), 3.91 (s, 1H), 3.86 (s, 3H), 1.73 (p, *J* = 6.0 Hz, 1H), 1.56 (s, 4H), 1.51–1.26 (m, 8H), 1.02–0.90 (m, 5H). Analysis calculated for C_19_H_22_Cl_2_O_2_S: C, 59.22; H, 5.75; S, 8.32. Found; C, 59.23; H, 5.74; S, 8.33%.

### 3.5. Molecular Docking Studies

#### 3.5.1. Methodology Section

**Figure 6:**
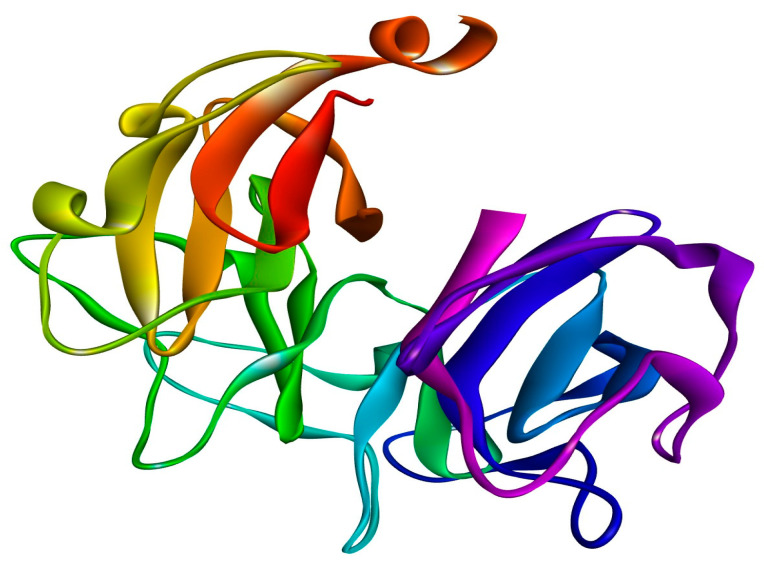
Crystal structure of the DNA gyrase of *Salmonella* Typhi with PDB ID: 5ztj, retrieved from the RCSB database.

Molecular docking studies were conducted for the synthesized compounds against bacterial enzyme PDB ID: 5ztj, which was retrieved from the RCSB database (https://www.rcsb.org/), accessed on 20 March 2024 and are presented in Figure 6 [31,32]. Before starting the docking studies, the structures of the synthesized compounds were drawn from the molecule structure builder tool of the 3D Molecular Operating Environment (MOE v2019). Subsequently, the energy of these protonated molecules was minimized by using the MMFF94x force field. The obtained database of ligands was used as an input file for the docking studies. On the other hand, the crystal structure of the targeted protein was also accomplished through MOE-protonated 3D tools. The water molecules were removed from the crystallographic structure of the targeted protein, and explicit hydrogen bonds were added by using the MOE. Auto-Dock Tools was used to calculate the atomic partial charges, and the synthesized compounds were docked into the active position of the targeted protein using Auto-Dock 4.2 with the standard parameter settings. Discovery Studio was used as a visualization tool, and high-scoring docked poses were considered as putative binding modes.

#### 3.5.2. Confirmation of the Isolates 

Blood samples of 5–10 mL were taken from a patient suspected of having septicemia, and ethical approval for the blood samples was obtained from the Ethical Review Committee (ERC), Government College University Faisalabad (Ref. No. GCUF/ERC/414). These samples were placed in a Bactec blood culture container and then inserted into the Bactec compact system (BioMérieux, Marcy-l′Étoile France) for up to five days. After the blood culture bottle proved positive, it was subcultured on *Salmonella* Shigella agar (SSA), and the plates were aerobically incubated at 37 °C nightly. The isolates were identified using bacterial morphology, Gram staining, oxidase activity, and biochemical reactions. Finally, the small Vitek 2 system, developed by BioMérieux in France, was used to confirm the strain biochemically.

#### 3.5.3. Antimicrobial Susceptibility Testing of XDR *S.* Typhi

Antimicrobial susceptibility testing was executed using a Vitek 2 compact system as per the CLSI guidelines. Chloramphenicol, ampicillin, co-trimoxazole, ceftriaxone, ciprofloxacin, azithromycin, imipenem, and meropenem were used. The results were interpreted as per the CLSI guidelines.

#### 3.5.4. Agar Well Diffusion Assay of Molecules **4A**–**4G** Compared to XDR *S.* Typhi Strain

The in vitro antibacterial activity of the freshly synthesized molecules **4A**–**4G** against XDR *S*. Typhi was evaluated using zone inhibition [33,34,35,36]. In brief, Mueller–Hinton agar plates were streaked with 0.5 McFarland bacterial suspension, and a sterile 6 mm cork borer was used to create wells in separate plates. Using sterile pipettes, different concentrations of the compounds **4A**–**4G** in each 100 DMSO solvent (10, 20, 30, 40, and 50 mg/mL) were independently put in the wells. The plates were then retained overnight for aerobic incubation at 37 °C. After a suitable incubation period, the zone of inhibition for each well was determined by using a vernier caliper. The experimental test was conducted three times, and the mean values for the ultimate antibacterial activity were determined (Table 2).

#### 3.5.5. Minimum Inhibitory Concentration of Molecules **4A**–**4G** against XDR S. Typhi

The MIC (mg/mL) of the aforementioned samples **4A**–**4G** was determined using the micro broth dilution test [37,38]. In a 50 mL Falcon tube, 2 to 3 individual colonies were introduced in a double-strength lysogeny broth (LB) medium having a 20 mL capacity and then hatched aerobically at 37 °C nightly. The bacterial interruption was subsequently diluted to achieve a 0.5 McFarland turbidity, corresponding to an optical density (OD) of 0.07 at 600 nm. In brief, serial dilutions of molecules **4A**–**4G** (5–50%) were organized in DMSO, and 100 µL of each compound dilution was distributed into ninety-six wells of a smooth bottom microtiter plate. Following this, 100 µL of bacterial interruption was added to a separate well. Negative-control wells were filled with 100 µL of LB, while positive-control wells were also filled with 100 µL of LB with bacterial suspension. The microtiter plate was then placed in a shaking hatchery (MaxQTM Mini 4450, Thermo Fisher Scientific Waltham MA, USA) and incubated overnight at 37 °C. MIC was determined by associating individual wells with both negative- and positive-control wells, with full measures conducted in triplicate.

#### 3.5.6. Minimum Bactericidal Concentration of Compounds **4A**–**4G** against XDR *S.* Typhi Strain

The first dilution that causes no progress on an agar plate is the lowest bactericidal concentration (mg/mL). A 10 µL taster was obtained from the microtiter plate’s no-visible-growth wells and injected onto nutrient agar plates (Oxoid, Hampshire, UK), where it was incubated aerobically at 37 °C ceaselessly to measure the MBC. The plates were examined for cell feasibility, and any bacterial or non-bacterial colonies that formed were noted. The concentration at which no discernible growth was observed was judged to be the MBC. The entire process was carried out three times [39].

## 4. Conclusions

This study was conducted on a 2-ethylhexyl 5-bromothiophene-2-carboxylate (**3**) synthesized through the Steglich esterification reaction by using commercially available 5-bromothiophene-2-carboxlic acid and 2-ethylhexanol. Compound **3** was arylated with numerous aryl boronic acids in this study by the Suzuki cross-coupling in the existence of Pd(PPh_3_)_4_ and K_3_PO_4_ to afford thiophene-based derivatives **4A**–**4G**. The antibacterial activity of compound **4F** inhibited the XDR *S*. Typhi clinical isolate at MIC 3.125 mg/mL due to the presence of thiophene moieties. Furthermore, the binding interactions of all produced compounds **4A**–**4G** with XDR *S*. Typhi proteins were theoretically assessed by molecular docking experiments to select the most active lead compounds. According to the molecular docking experiments, chemicals attach to the XDR S. Typhi proteins, showing that they are active against biological targets. In addition, **4F** showed the highest binding free energy (−7.38549 KCal/mole) compared to the standard reference drug ciprofloxacin (−5.61799 KCal/mole). This study will help to develop new therapeutic drugs to treat the infections produced by XDR S. Typhi pathogens after several clinical trials.

## Figures and Tables

**Table 1:** Percentage yield of the derivatives of 2-ethylhexyl 5-bromothiophene-2-carboxylate (**4A**–**4G**).

Entry	Aryl Boronic Acid	Product	Solvent	Yield
**4A**	4-MeOC_6_H_4_B(OH)_2_	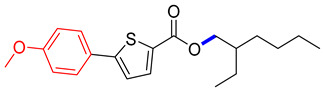	1,4-dioxane	79%
**4B**	3,5-Me_2_C_6_H_3_B(OH)_2_	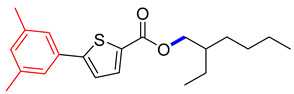	1,4-dioxane	74%
**4C**	4-ClC_6_H_4_B(OH)_2_	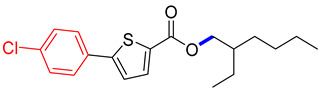	1,4-dioxane	65%
**4D**	3-Cl,4-FC_6_H_3_B(OH)_2_	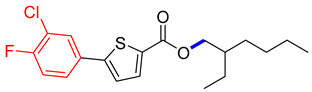	1,4-dioxane	60%
**4E**	4-MeC_6_H_4_B(OH)_2_	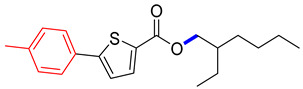	1,4-dioxane	72%
**4F**	C_4_H_3_B(OH)_2_S	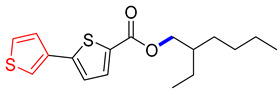	1,4-dioxane	69%
**4G**	3,4-Cl_2_C_6_H_3_B(OH)_2_	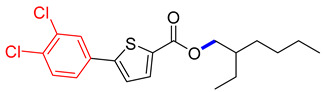	1,4-dioxane	62%

**Table 2:** MIC and MBC (mg/mL) values of molecules **4A**–**4G** compared to XDR *Salmonella* Typhi.

Entry No.	MIC (mg/mL)	MBC (mg/mL)	Ciprofloxacin (µg/mL)	Ceftriaxone (µg/mL)
4A	12.5	25	≥1	≥4
4B	25	50	≥1	≥4
4C	3.125	6.25	≥1	≥4
4D	12.5	25	≥1	≥4
4E	6.25	12.5	≥1	≥4
4F	3.125	6.25	≥1	≥4
4G	25	50	≥1	≥4

**Table 3:** Zone of inhibition (mm) of molecules **4A**–**4G** compared to XDR *Salmonella* Typhi.

Entry No.	Zone mm (50 mg)	Zone mm (40 mg)	Zone mm (30 mg)	Zone mm (20 mg)	Zone mm (10 mg)	Ciprofloxacin (5 µg)	Ceftriaxone (30 µg)
**4A**	17	13	12	12	11	<20	<19
**4B**	4	4	4	4	4	<20	<19
**4C**	15	15	13	12	11	<20	<19
**4D**	16	15	13	12	11	<20	<19
**4E**	4	4	4	4	4	<20	<19
**4F**	25	23	23	21	23	<20	<19
**4G**	12	10	10	10	18	<20	<19

**Table 4:** Interactions and binding free energy of the synthesized 2-ethylhexyl 5-bromothiophene-2-carboxylate’s derivatives (**4A**–**4G**).

Compound	Interactions	Free Energy ΔG (KCal/mole)
**4A**	H bonding: Lys550 C–H interaction: Arg612 π–σ interaction: Arg615 Alkyl and π-alkyl interaction: Tyr548, Ile578, Val839	−6.23269
**4B**	H bonding: Lys550 C–H interaction: Arg612 Alkyl and π-alkyl interaction: Val540, Tyr548, Tyr557, Ile578	−6.99511
**4C**	H bonding: Lys550 Alkyl and π-alkyl interaction: Ile578, Arg612, Arg615, Val839	−5.93047
**4D**	H bonding: Lys550, Asp576 C–H interaction: Thr542 π–anion interaction: Asp576 Amide-π stacked: Gly613 Alkyl and π-alkyl interaction: Tyr557, Ile578, Val839	−7.12247
**4E**	H bonding: Lys550 C–H interaction: Arg612 π–anion interaction: Asp576 Alkyl and π-alkyl interaction: Arg615, Val839	−6.11878
**4F**	H bonding: Lys550, Arg612 π-sulfur: Tyr548 Alkyl and π-alkyl interaction: Ile578, Leu581, Arg615, Val839	−7.38549
**4G**	H bonding: Gln560 C–H interaction: Lys550 Amide-π stacked: Gly613 Alkyl and π-alkyl interaction: Tyr548, Ile578, Arg612, Val839	−6.97763
Ciprofloxacin	H bonding: Lys550, Arg612, Gly613 C–H interaction: Tyr557, Asp576, Arg615 Alkyl and π-alkyl interaction: Ile578, Arg615	−5.61799

**Table 5:** Pharmacokinetic studies based on the in silico method (SwissADME).

Code	TPSA ^a^	Lipinski Violation	PAINS ^b^	MLOGP ^c^	NRB ^d^	HBD ^e^	GI Absorption ^f^	HBA ^g^
**4A**	63.77	0	0	4.80	10	0	High	3
**4B**	54.54	0	0	4.41	9	0	High	2
**4C**	54.54	0	0	4.44	9	0	High	2
**4D**	54.54	0	0	4.42	9	0	Low	3
**4E**	54.54	0	0	4.51	9	0	High	2
**4F**	82.78	0	0	3.85	9	0	High	2
**4G**	54.54	1	0	5.27	9	0	Low	2

^a^ Topological polar surface area, ^b^ Pan-assay interference, ^c^ Logarithm of partition coefficient between *n*-octanol and water, ^d^ Number of rotatable bonds, ^e^ Hydrogen bond donor, ^f^ Gastrointestinal absorption, ^g^ Hydrogen bond acceptor.

**Table 6:** Energies of HOMO, LUMO, and the energy difference between HOMO–LUMO.

Compounds	E_HOMO_ (eV)	E_LUMO_ (eV)	Difference (eV)	Hyperpolarizability
**4A**	−5.98	−1.63	4.34	4644.46
**4B**	−6.33	−1.71	4.61	1833.79
**4C**	−6.42	−1.85	4.56	2566.26
**4D**	−6.55	−1.85	4.69	1878.87
**4E**	−6.18	−1.85	4.33	2379.28
**4F**	−6.25	−1.67	4.57	2413.44
**4G**	−6.55	−1.96	4.59	2184.60
Ciprofloxacin	−5.98	−1.18	4.80	2796.36

**Table 7:** *I*, *A*, *η*, *µ*, and *ω* of the molecules under study.

Comp. No.	*I* **(eV)**	*A* **(eV)**	*η* **(eV)**	*µ* **(eV)**	*ω* **(eV)**
**4A**	5.98	1.63	2.17	−3.81	3.34
**4B**	6.33	1.71	2.30	−4.02	3.51
**4C**	6.42	1.85	2.28	−4.13	3.74
**4D**	6.55	1.85	2.34	−4.20	3.76
**4E**	6.18	1.85	2.16	−4.02	3.73
**4F**	6.25	1.67	2.28	−3.96	3.43
**4G**	6.55	1.96	2.29	−4.26	3.95

## Data Availability

Data are contained within the article and the supplementary materials.

## Supplementary Materials

The following supporting information can be downloaded at: https://www.mdpi.com/article/10.3390/molecules29133005/s1, Figure S1: ^1^H NMR spectrum of compound **4A**; Figure S2: ^1^H NMR spectrum of compound **4D**; Figure S3: ^1^H NMR spectrum of compound **4E**; Figure S4: ^1^H NMR spectrum of compound **4F**; Figure S5: Antibacterial activity of compounds (**4A**–**4G**) against XDR *Salmonella* Typhi; Figure S6: The putative binding mode of **4A** within the active pocket of DNA gyrase protein; Figure S7: The putative binding mode of **4B** within the active pocket of DNA gyrase protein; Figure S8: The putative binding mode of **4C** within the active pocket of DNA gyrase protein; Figure S9: The putative binding mode of **4D** within the active pocket of DNA gyrase protein; Figure S10: The putative binding mode of **4E** within the active pocket of DNA gyrase protein; Figure S11: The putative binding mode of **4G** within the active pocket of DNA gyrase protein; Figure S12: Optimized geometries of all the synthesized molecules (**4A**–**4G**); Table S1: Comparison of experimental and theoretically calculated ^1^H-NMR data for **4A**; Table S2: Comparison of experimental and theoretically calculated ^1^H-NMR data for **4B**; Table S3: Comparison of experimental and theoretically calculated ^1^H-NMR data for **4C**; Table S4: Comparison of experimental and theoretically calculated ^1^H-NMR data for **4D**; Table S5: Comparison of experimental and theoretically calculated ^1^H-NMR data for **4E**; Table S6: Comparison of experimental and theoretically calculated ^1^H-NMR data for **4F**; Table S7: Comparison of experimental and theoretically calculated ^1^H-NMR data for **4G**.
