# Supplementary material for: Synthesis of 2-Ethylhexyl 5-Bromothiophene-2-Carboxylates; Antibacterial Activities against Salmonella Typhi, Validation via Docking Studies, Pharmacokinetics, and Structural Features Determination through DFT
Source: Molecules. 2024 Jun 25;29(13):3005. doi: 10.3390/molecules29133005 (PMC11242937; doi:10.3390/molecules29133005)

Supplementary Data

Synthesis of 2-Ethylhexyl 5-Bromothiophene-2-Carboxylates; Antibacterial Activities against *Salmonella* Typhi, Validation via Docking Studies, Pharmacokinetics, and Structural Features Determination through DFT

Waseem Nazeer [1], Muhammad Usman Qamar [2,3], Nasir Rasool [1,*], Mohamed Taibi[4], Ahmad Mohammad Salamatullah[5]

[1] Department of Chemistry, Government College University, Faisalabad 38000, Pakistan; waseemgondal78@gmail.com (W.N.); nasirrasool@gcuf.edu.pk (N.R.);

[2] Institute of Microbiology, Government College University, Faisalabad 38000, Pakistan; musmanqamar@gcuf.edu.pk (M.U.Q.);

3 Division of Infectious Disease and Department of Medicine, University of Geneva, Geneva, 206, Switzerland.

4 Laboratory of Therapeutic and Organic Chemistry, Faculty of Pharmacy, University of Montpellier, Montpellier, 34000 France Mohamedtaibi9@hotmail.fr (M.T.);

[5] Department of Food Science & Nutrition, College of Food and Agricultural Sciences, King Saud University, 11 P.O. Box 2460, Riyadh 11451, Saudi Arabia; asalamh@ksu.edu.sa (A.M.S.);

* Corresponding author's E-mail addresses:

Nasir Rasool Email: nasirrasool@gcuf.edu.pk

Table of Contents:

Figure S1: ^{1}H NMR spectrum of compound **4A** 3

Figure S2: ^{1}H NMR spectrum of compound **4D** 3

Figure S3: ^{1}H NMR spectrum of compound **4E** 4

Figure S4: ^{1}H NMR spectrum of compound **4F** 4

Figure S5: Antibacterial activity of compounds (**4A-4G**) against XDR *Salmonella* Typhi 5

Figure S6: The putative binding mode of **4A** within the active pocket of DNA gyrase protein 6

Figure S7: The putative binding mode of **4B** within the active pocket of DNA gyrase protein 6

Figure S8: The putative binding mode of **4C** within the active pocket of DNA gyrase protein 7

Figure S9: The putative binding mode of **4D** within the active pocket of DNA gyrase protein 7

Figure S10: The putative binding mode of **4E** within the active pocket of DNA gyrase protein 8

Figure S11: The putative binding mode of **4G** within the active pocket of DNA gyrase protein 8

Figure S12: Optimized geometries of all the synthesized molecules (**4A-4G**) 9

Table S1: Comparison of experimental and theoretically calculated ^{1}H-NMR data for **4A** 10

Table S2: Comparison of experimental and theoretically calculated ^{1}H-NMR data for **4B** 11

Table S3: Comparison of experimental and theoretically calculated ^{1}H-NMR data for **4C** 12

Table S4: Comparison of experimental and theoretically calculated ^{1}H-NMR data for **4D** 13

Table S5: Comparison of experimental and theoretically calculated ^{1}H-NMR data for **4E** 14

Table S6: Comparison of experimental and theoretically calculated ^{1}H-NMR data for **4F** 15

Table S7: Comparison of experimental and theoretically calculated ^{1}H-NMR data for **4G** 16

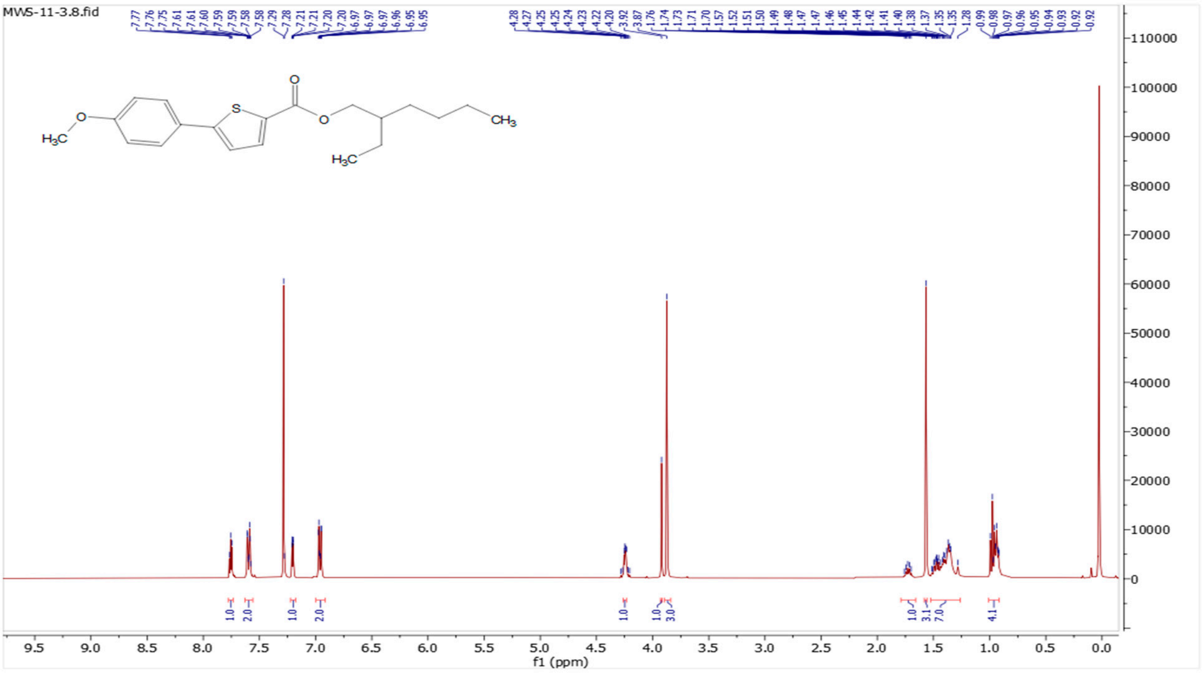

Figure S1: ^{1}H NMR spectrum of compound **4A**

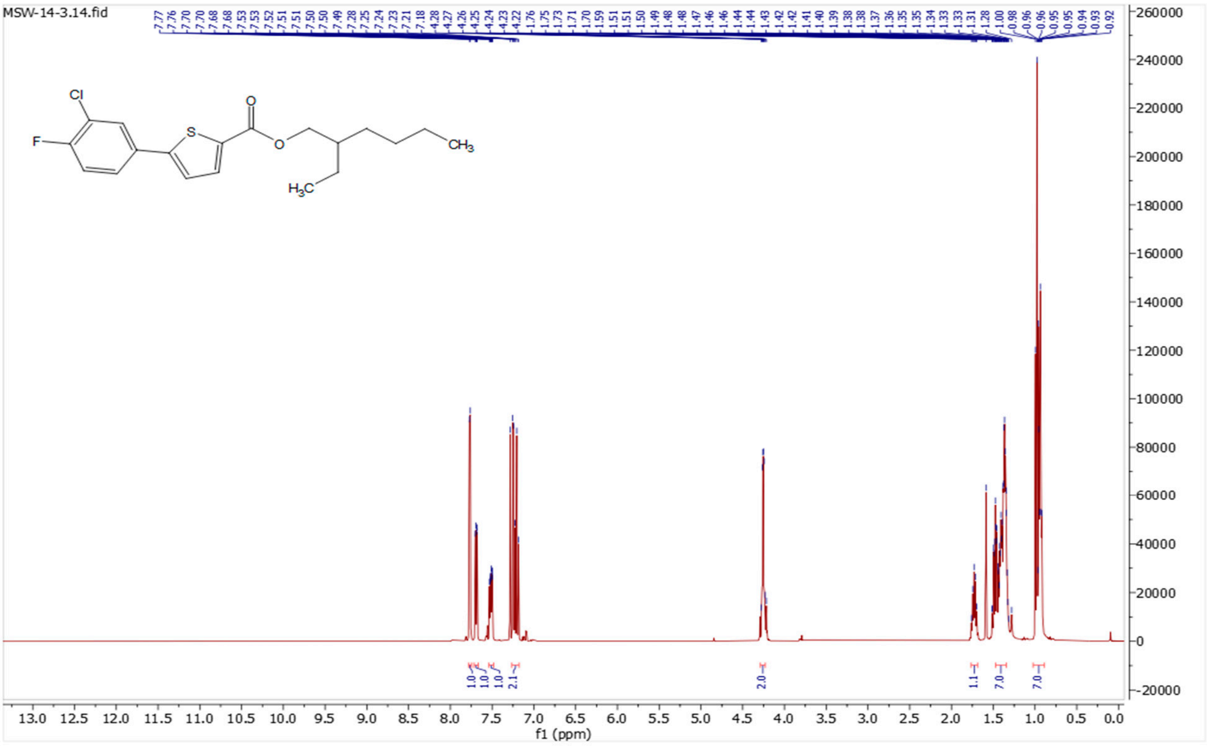

Figure S2: ^{1}H NMR spectrum of compound **4D**

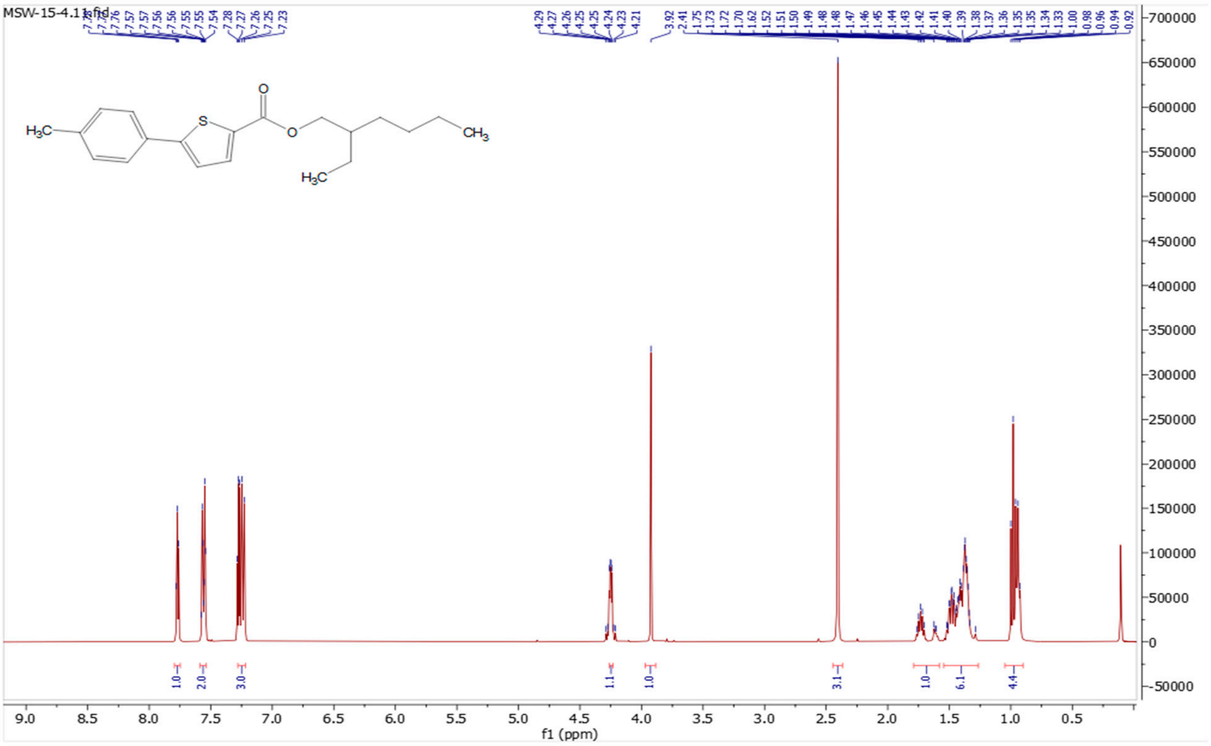

Figure S3: ^{1}H NMR spectrum of compound **4E**

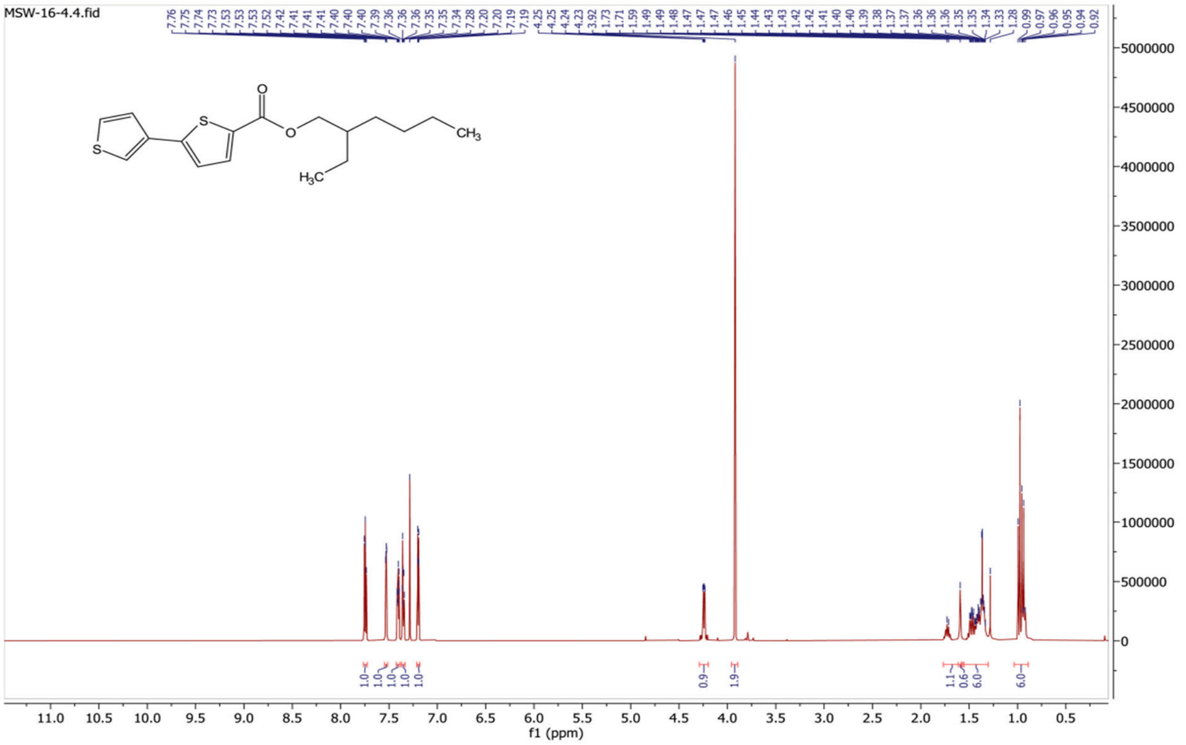

Figure S4: ^{1}H NMR spectrum of compound **4F**

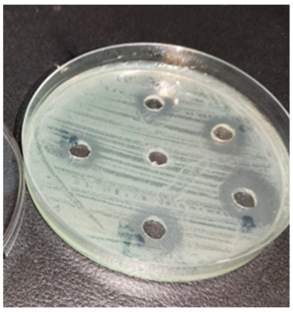

4A

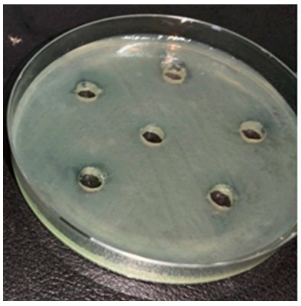

4B

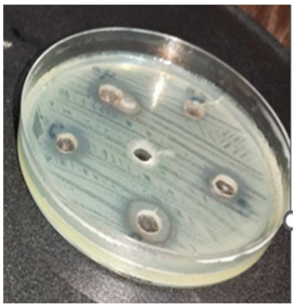

4C

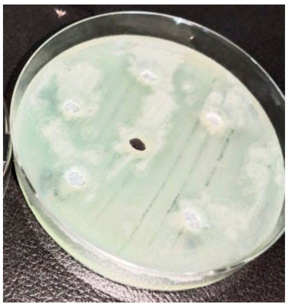

4D

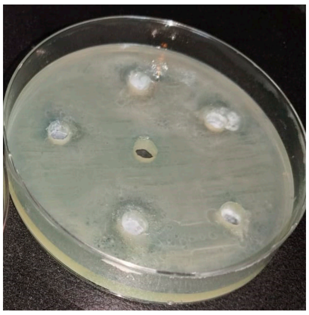

4E

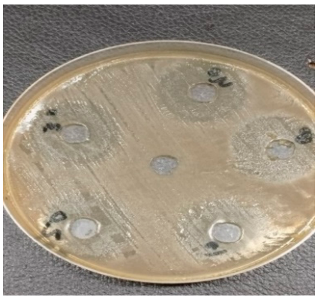

4F

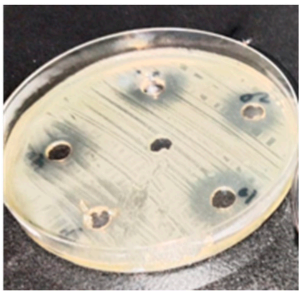

4G

Figure S5: Antibacterial activity of compounds (**4A-4G**) against XDR *Salmonella* Typhi

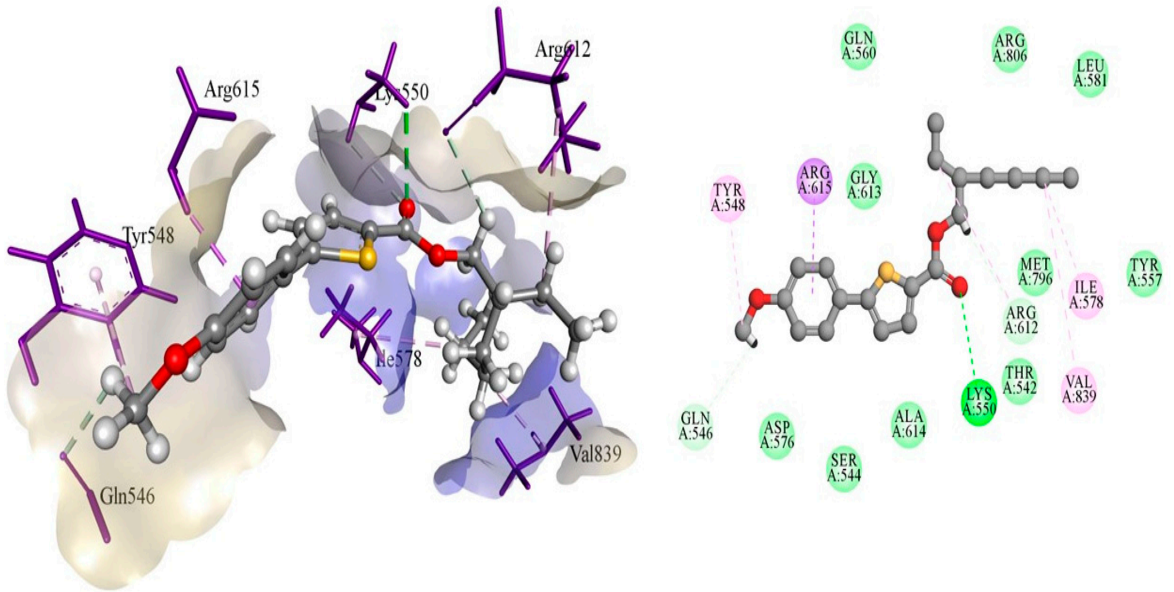

Figure S6: The putative binding mode of **4A** within the active pocket of DNA gyrase protein PDB ID: **5ztj**

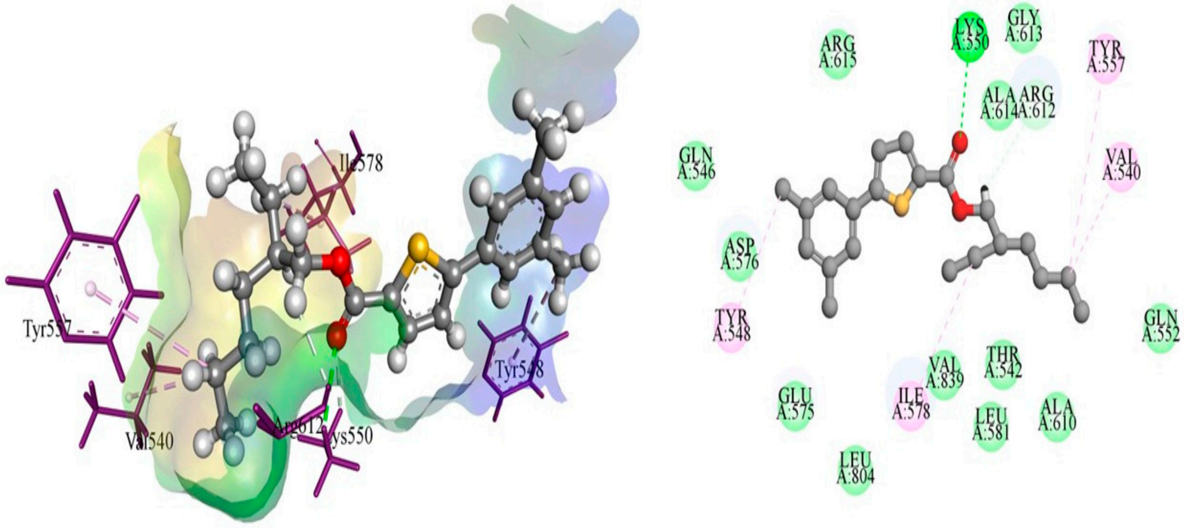

Figure S7: The putative binding mode of **4B** within the active pocket of DNA gyrase protein PDB ID: **5ztj.**

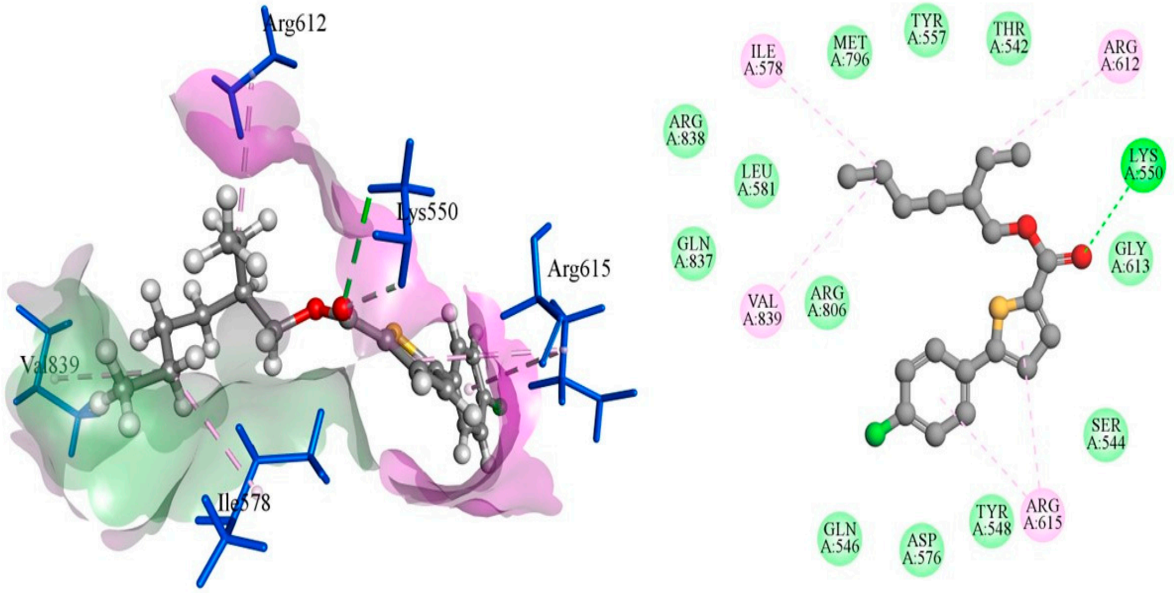

Figure S8: The putative binding mode of **4C** within the active pocket of DNA gyrase protein PDB ID: **5ztj.**

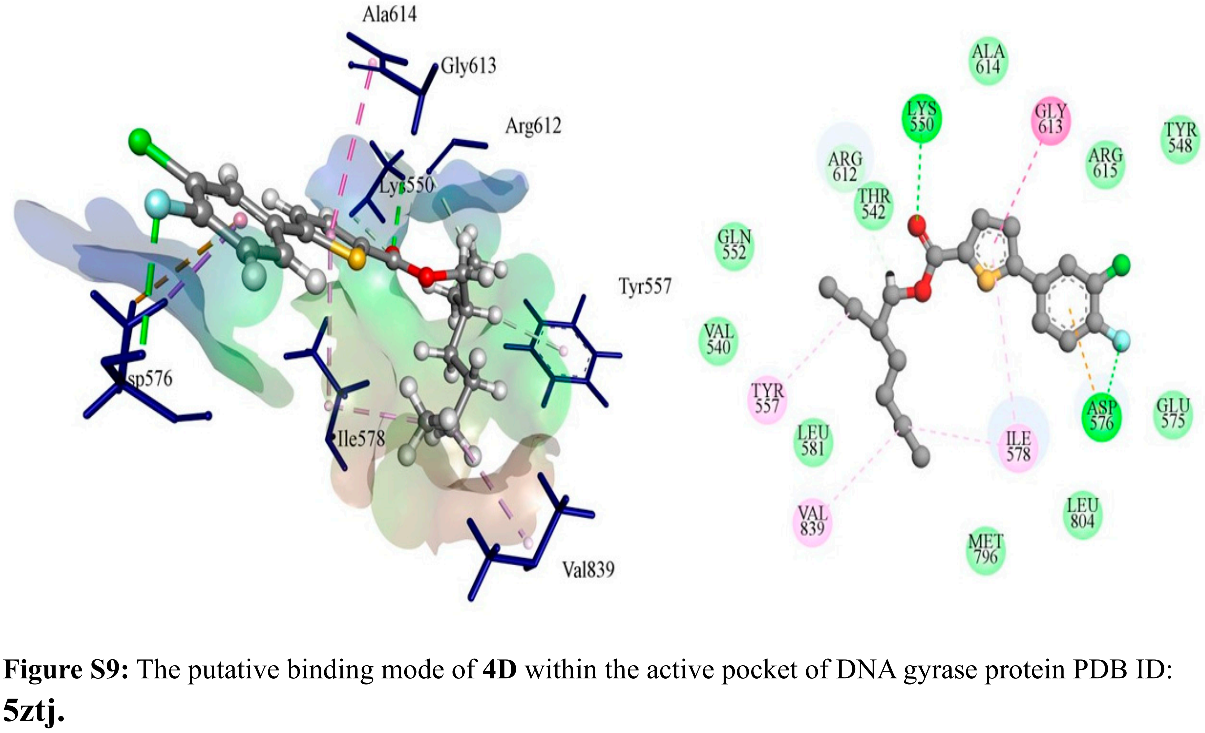

Figure S9: The putative binding mode of **4D** within the active pocket of DNA gyrase protein PDB ID: **5ztj.**

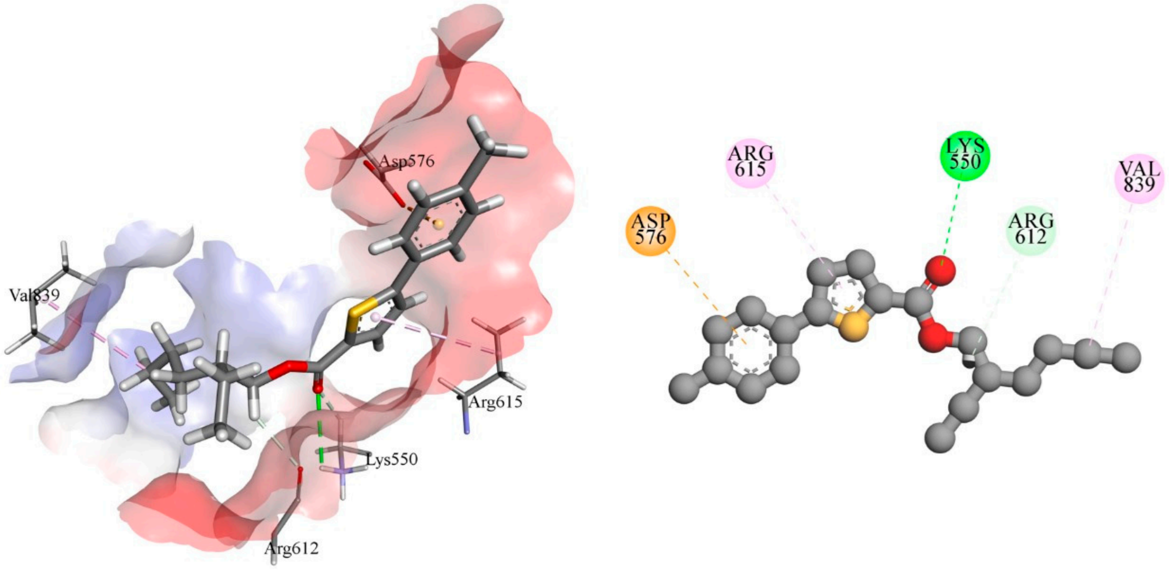

Figure S10: The putative binding mode of **4E** within the active pocket of DNA gyrase protein PDB ID: **5ztj.**

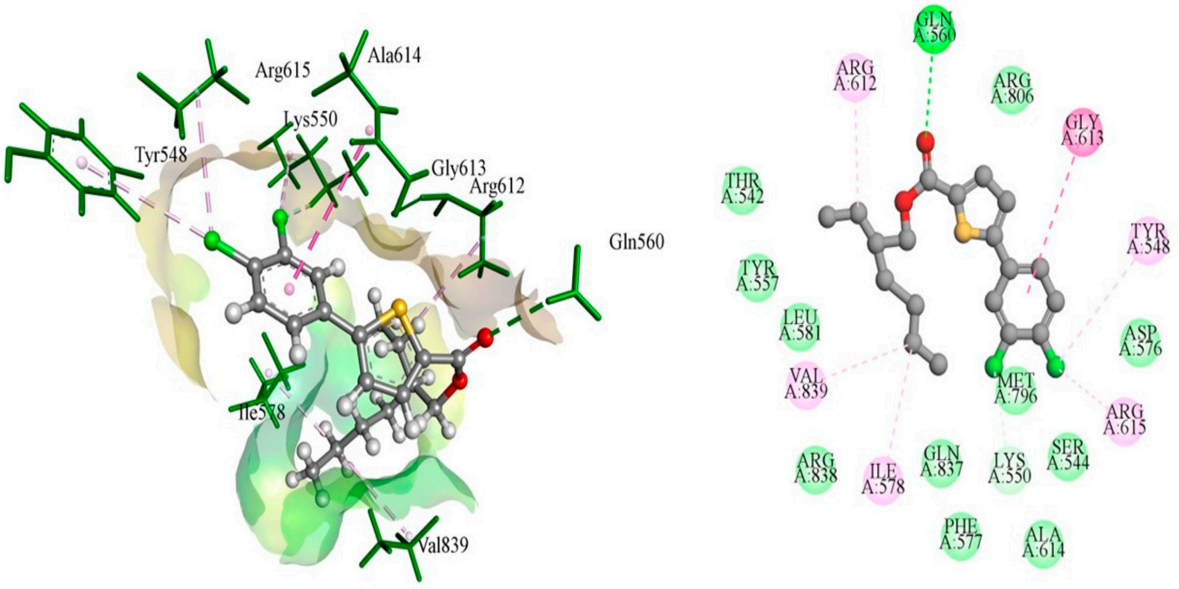

Figure S11: The putative binding mode of **4G** within the active pocket of DNA gyrase protein PDB ID: **5ztj.**

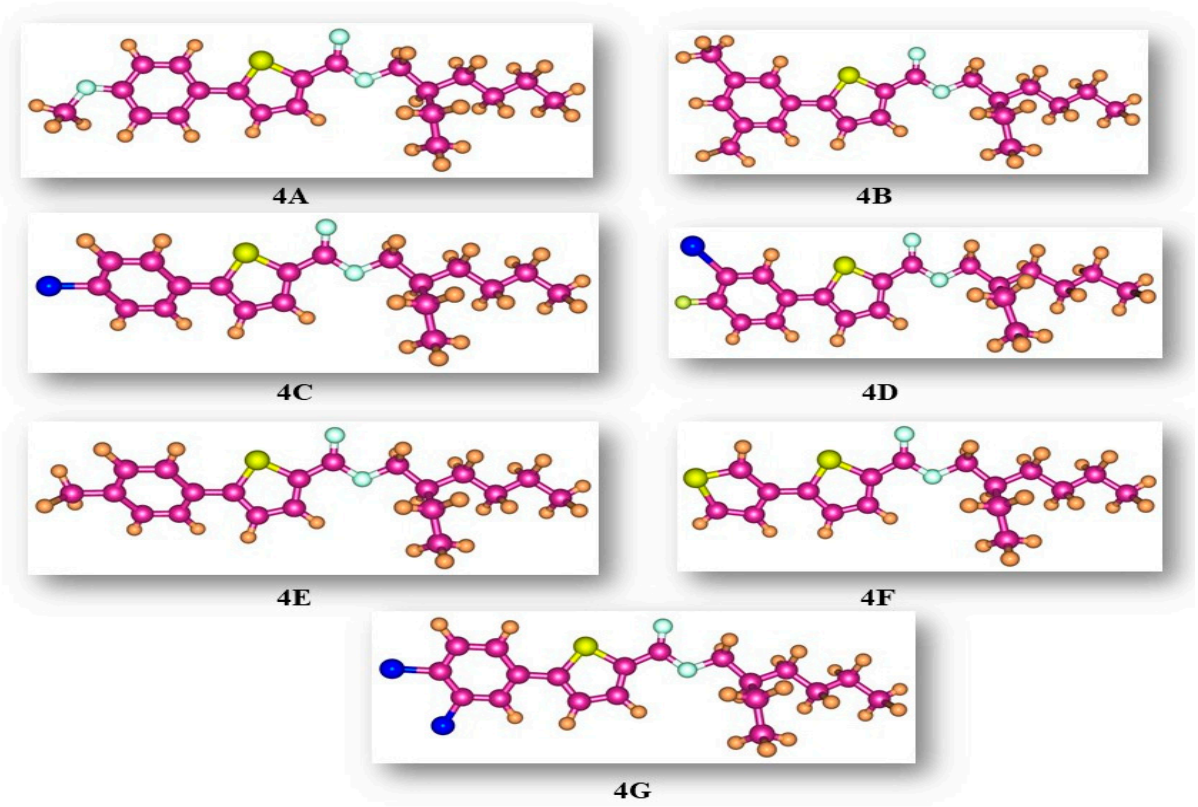

Figure S12: Optimized geometries of all the synthesized molecules (4A-4G).

Table S1: Comparison of experimental and theoretically calculated ^{1}H-NMR data for **4A**

**4A**			
**Carbon Number**	**Carbon Type**	**^{1}H-NMR (Exp) δ, ppm**	**^{1}H-NMR (Cal) δ, ppm**
3	CH	7.68	7.10
4	CH	7.83	7.57
7	CH	7.68	7.60
8	CH	7.03	6.91
10	CH	7.03	6.60
11	CH	7.68	7.67
15	CH2	4.06-4.32	3.53
16	CH	1.67	1.34
17	CH2	1.30-1.50	1.04
18	CH2	1.30	1.25
19	CH2	1.30	1.11
20	CH3	0.89	0.94
21	CH2	1.30-1.50	1.19
22	CH3	0.89	0.96
24	CH3	3.80	3.54

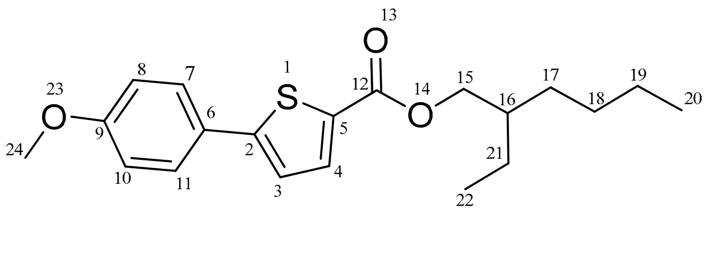

Table S2: Comparison of experimental and theoretically calculated ^{1}H-NMR data for **4B**

**4B**			
**Carbon Number**	**Carbon Type**	**^{1}H-NMR (Exp) δ, ppm**	**^{1}H-NMR (Cal) δ, ppm**
3	CH	7.37	7.60
4	CH	7.82	7.25
7	CH	7.37	7.33
9	CH	6.96	6.95
11	CH	7.37	7.29
15	CH_2	4.06, 4.32	3.54
16	CH	1.67	1.31
17	CH_2	1.30, 1.49	1.05
18	CH_2	1.30	0.79
19	CH_2	1.30	1.11
20	CH_3	0.89	0.81
21	CH_2	1.30, 1.49	1.29
22	CH_3	0.89	0.97
23	CH_3	2.30	2.09
24	CH_3	2.30	2.09

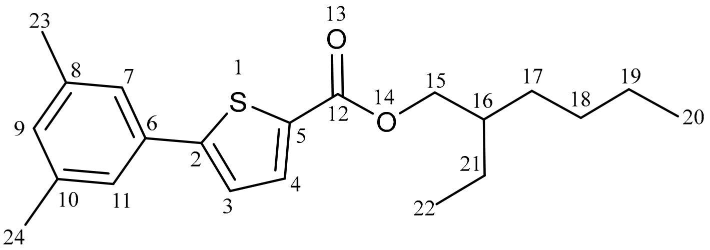

Table S3: Comparison of experimental and theoretically calculated ^{1}H-NMR data for **4C**

**4C**			
**Carbon Number**	**Carbon Type**	**^{1}H-NMR (Exp) δ, ppm**	**^{1}H-NMR (Cal) δ, ppm**
3	CH	7.67	7.15
4	CH	7.83	7.63
7	CH	7.67	7.65
8	CH	7.40	6.95
10	CH	7.40	6.48
11	CH_2	7.67	7.34
15	CH_2	4.06, 4.32	3.76
16	CH	1.67	1.33
17	CH_2	1.30, 1.50	1.04
18	CH_2	1.30	1.29
19	CH_2	1.30	1.15
20	CH_3	0.89	0.96
21	CH_2	1.30	1.18
22	CH_3	0.89	0.96

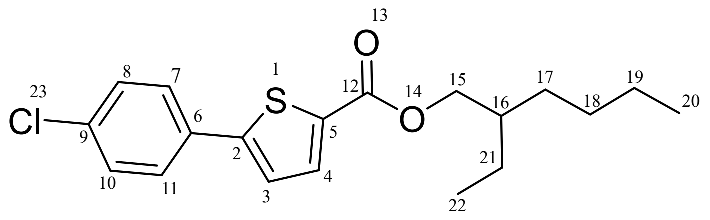

Table S4: Comparison of experimental and theoretically calculated ^{1}H-NMR data for **4D**

**4D**			
**Carbon Number**	**Carbon Type**	**^{1}H-NMR (Exp) δ, ppm**	**^{1}H-NMR (Cal) δ, ppm**
3	CH	7.77	7.15
4	CH	7.69	7.63
7	CH	7.26	7.65
10	CH	7.51	6.95
11	CH	7.17	6.48
15	CH_2	4.41-4.10	7.34
16	CH	1.73	3.76
17	CH_2	1.49-1.34	1.33
18	CH_2	1.49-1.34	1.04
19	CH_2	1.49-1.34	1.29
20	CH_3	1.02-.091	1.15
21	CH_2	0.91-1.49	0.96
22	CH_3	1.02-.091	1.18

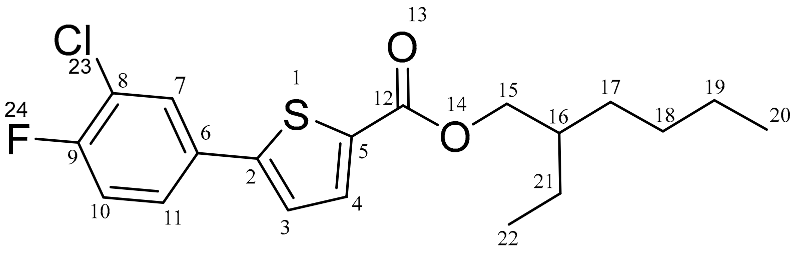

Table S5: Comparison of experimental and theoretically calculated ^{1}H-NMR data for **4E**

**4E**			
**Carbon Number**	**Carbon Type**	**^{1}H-NMR (Exp) δ, ppm**	**^{1}H-NMR (Cal) δ, ppm**
3	CH	7.22	7.58
4	CH	7.77	7.27
7	CH	7.56	7.35
8	CH	7.28	6.98
10	CH	7.28	7.27
11	CH	7.56	7.29
15	CH_2	4.30-4.16	3.60
16	CH	1.83-1.59	1.34
17	CH_2	1.53-1.25	1.09
18	CH_2	1.53-1.25	0.83
19	CH_2	1.53-1.25	1.15
20	CH_3	0.96	0.84
21	CH_2	1.53-1.25	1.31
22	CH_3	0.96	0.97
23	CH_3	2.41	2.08

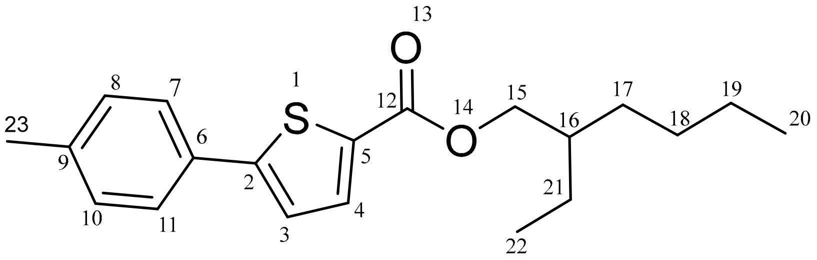

Table S6: Comparison of experimental and theoretically calculated ^{1}H-NMR data for **4F**

**4F**			
**Carbon Number**	**Carbon Type**	**^{1}H-NMR (Exp) δ, ppm**	**^{1}H-NMR (Cal) δ, ppm**
3	CH	7.20	7.24
4	CH	7.74	7.32
7	CH	7.41	7.65
8	CH	7.35	6.91
10	CH	7.53	6.97
14	CH_2	3.92	3.77
15	CH	1.77-1.57	1.33
16	CH_2	1.54-1.28	1.09
17	CH_2	1.54-1.28	1.04
18	CH_2	1.54-1.28	1.29
19	CH_3	0.96	1.13
20	CH_2	1.54-1.28	0.96
21	CH_3	0.96	1.14

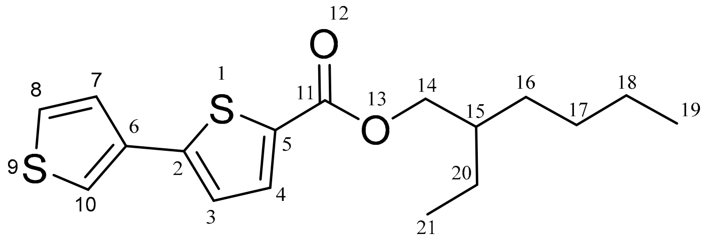

Table S7: Comparison of experimental and theoretically calculated ^{1}H-NMR data for **4G**

**4G**			
**Carbon Number**	**Carbon Type**	**^{1}H-NMR (Exp) δ, ppm**	**^{1}H-NMR (Cal) δ, ppm**
3	CH	7.04-6.90	7.35
4	CH	7.75	7.57
7	CH	7.65-7.53	7.67
10	CH	7.20	6.91
11	CH	7.65-7.53	6.58
15	CH_2	4.24, 3.92	7.34
16	CH	1.73	1.48
17	CH_2	1.51-1.26, 1.57	1.37
18	CH_2	1.51-1.26	1.04
19	CH_2	1.51-1.26	1.37
20	CH_3	1.01-0.90	1.09
21	CH_2	1.51-1.26, 1.57	0.97
22	CH_3	1.01-0.90	1.19

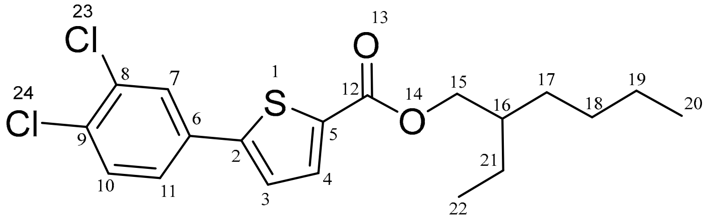

Supplement: Supplementary file 1 [file molecules-29-03005-s001.zip › molecules-2969330-supplementary.pdf]
